# Depressive and anxiety symptoms among Japanese cancer survivors: Japan cancer survivorship research project

**DOI:** 10.1186/s12885-022-09215-x

**Published:** 2022-02-02

**Authors:** Motoki Endo, Kentaro Matsui, Rie Akaho, Kiyomi Mitsui, Yan Yan, Yuya Imai, Yuito Ueda, Go Muto, Gautam A. Deshpande, Yasuhisa Terao, Satoru Takeda, Mitsue Saito, Kazuhiko Hayashi, Katsuji Nishimura, Takeshi Tanigawa

**Affiliations:** 1 Department of Public Health, Juntendo University Faculty of Medicine, Tokyo, Japan; 2 Department of Psychiatry, Tokyo Women’s Medical University School of Medicine, Tokyo, Japan; 3 Department of Clinical Laboratory and Department of Sleep-Wake Disorders, National Center of Neurology and Psychiatry, National Institute of Mental Health, Kodaira, Japan; 4 Department of Hygiene, Public Health, and Preventive Medicine, Showa University, Tokyo, Japan; 5 Department of Palliative Medicine, Juntendo University Graduate School of Medicine, Tokyo, Japan; 6 Department of Hygiene, Kitasato University School of Medicine, Kanagawa, Japan; 7 Department of General Medicine, Juntendo University Graduate School of Medicine, Tokyo, Japan; 8 Department of Obstetrics and Gynecology, Juntendo University Faculty of Medicine, Tokyo, Japan; 9 Department of Breast Oncology, Juntendo University School of Medicine, Tokyo, Japan; 10 St. Marianna University School of Medicine, Kawasaki, Japan

**Keywords:** Depressive symptoms, Anxiety, Cancer survivors, HADS

## Abstract

**Background:**

This study aimed to clarify predictors of depressive symptoms and anxiety symptoms after cancer diagnosis among Japanese cancer survivors (CSs).

**Methods:**

As part of a Japanese cancer survivorship research project commissioned by the Ministry of Health, Labour and Welfare (MHLW) of Japan, we conducted a web-based nationwide survey of CSs in 2018. We analyzed the risk factors for depressive and anxiety symptoms, as measured by the Hospital Anxiety and Depression Scale Japanese version (HADS).

**Results:**

Of 1,234 Japanese CSs, mean score of HADS-depression and HADS-anxiety were 4.08 and 4.78, respectively. At the time of the study, the number of CSs with symptoms of depression and anxiety were 111 (9.0%) and 269 (21.8%), respectively. After multivariable analysis, CSs ≥ 60 years old (reference: ≤ 39 years old, odds ratios (OR): 0.39, 95%CI: 0.17–0.90) and those ≥ 10 years from cancer diagnosis (reference: 0–4 years, OR: 0.55, 95%CI: 0.32–0.96) had lower odds for depressive symptoms. And CSs ≥ 60 years old (reference: ≤ 39 years old, OR: 0.27, 95%CI: 0.15–0.49) and those ≥ 10 years from cancer diagnosis (reference: 0–4 years, OR: 0.62, 95%CI: 0.42–0.90) also had lower odds for anxiety symptoms. CSs who received chemotherapy (OR: 1.56, 95%CI: 1.10–2.20) had higher odds for anxiety symptoms.

**Conclusions:**

Based on manifestation of symptoms, CSs who were younger, closer to the time of cancer diagnosis, had advanced-staged cancer, or received chemotherapy may be at higher risk for depressive or anxiety symptoms. Those CSs who have higher risk for depression and anxiety symptoms, should be followed-up more carefully for better cancer survivorship, by medical professionals, companies, and society.

## Background

Cancer survivors (CSs) experience a wide variety of symptoms due to both the pathophysiology of cancer as well as its treatments [1]. Many CSs also experience symptoms of depression and anxiety during their survivorship, including concerns regarding fear of recurrence, fear of death, complicated and psychologically and physically stressful treatment regimens, and the restriction of daily activities [2]. Depressive symptoms (including persistent low mood, loss of interest, fatigue, feelings of worthlessness) and anxiety symptoms (including worry, restlessness, poor sleep, somatic symptoms), the two of which commonly occur together, are frequently reported as psychological symptoms among CSs [3]. Mitchell’s meta-analysis reported that prevalence of self-reported depression and anxiety among approximately 50,000 CSs from developed countries as about 12% and 18%, respectively [4]. World Mental Health Japan Survey reported in 2016 that 12-month prevalence of mood disorders and anxiety disorders in Japanese general populations was 2.3%, and 4.9%, respectively[5]. Both depressive and anxiety symptoms have been reported to have profoundly negative effects on CSs, including reduced quality of life and greater rates of suicide [6]. Despite this, depression and anxiety often goes under-detected by medical professionals in the clinical oncology setting[7]. As such, routine screening instruments like the Hospital Anxiety and Depression Scale (HADS) may be useful tools to facilitate the early detection of depression or anxiety among CSs[8].

To our knowledge, little is known about predictors of depressive and anxiety symptoms among Japanese CSs. The objective of this study was to clarify the predictors of depressive and anxiety symptoms among Japanese CSs of various types of cancer. Clarifying the risk of depressive and anxiety symptoms among CSs may help healthcare providers identify at-risk groups and better support the mental and physical well-being of CSs.

## Methods

A web-based survey was conducted targeting CSs using data from a 2018 Japanese cancer survivorship research project (Endo-Han) commissioned by the Ministry of Health, Labour and Welfare (MHLW) of Japan, In November 2018, a web-based questionnaire survey was conducted by Macromill Inc. (www.macromill.com/global/index.html), a leading Internet research company in Japan, who sent questionnaires to all 11,562 registered CSs, aged 18 − 65 years, while in this project published previously [9, 10]. Of these, 1,610 completed the questionnaire (response rate: 13.9%). In addition to collecting routine demographic data, the questionnaire collected additional clinically-relevant variables including age at cancer diagnosis; year of initial cancer diagnosis; cancer stage; cancer site; medical history; experience with cancer therapy, including surgery, chemotherapy, and radiotherapy. Of the 1,610 respondents, we excluded the following participants: 192 who were diagnosed with cancer for the first time before 2000 (as cancer treatment improved dramatically after that time in Japan); 107 who had a psychiatric diagnosis prior to cancer diagnosis (*n* = 107); 60 who had more than two kinds of cancers (confounding comparison between cancer types); and 17 for whom no cancer data were available. Data from 1,234 CSs was analyzed in this study.

As for statistical methods, a multivariable logistic regression model was used to analyze the predictors of multivariable logistic regression was used to obtain odds ratios with 95% confidence intervals for the relative odds of depressive and anxiety symptoms. The Japanese version of the HADS (HADS-J) was validated among Japanese CSs by Kugaya et al.[11]. The optimal cut-off point of HADS-J-Depression, Anxiety was 10/11, 7/8, respectively, for screening major depressive disorder[11]. Subjects were dichotomized by these cut-off points, which meant that those with scores less than 10 were categorized as “less depressive group,” with those having scores more than 11 categorized as the “more depressive group.” For anxiety symptoms, those in the “lower anxiety group” had score less than 7, while the “higher anxiety group” had scores more than 8. All analyses were conducted using IBM SPSS Statistics for Windows (version 25.0; IBM Corp, Armonk, NY, USA). The medical ethics committee of Juntendo University informed us that after reading the purpose of this research project, answering this questionnaire meant consent to this research, in accordance with national guidelines [12]. This study was approved by the medical ethics committee of Juntendo University (ethical number: 2017067). The data that support the findings of this study could be available on request from the corresponding author. The data are not publicly available due to privacy or ethical restrictions.

## Results

In total, 1,234 Japanese patients diagnosed with cancer between 2001 and 2018 were enrolled in the study, details of which are shown in Table 1. The characteristics of the subjects were as follows: 690 (55.9%) of participants were men (mean (± SD) age at cancer diagnosis, 56.14 (± 8.18) years old), and 544 (44.1%) were women (mean age at diagnosis, 45.51 (± 9.29)). Regarding cancer type, breast cancer survivors were most represented in the sample (*n* = 230, 18.6%), followed by intestinal CSs (*n* = 199), which included colorectal cancer (*n* = 191, 16.1%), small intestine cancer (*n* = 4, 0.3%), and appendix cancer (*n* = 4, 0.3%). Female genital cancers (*n* = 152, 12.3%) included uterine (*n* = 136, 11.0%) and ovarian (*n* = 16, 1.3%) cancers. Male genital cancers (*n* = 111, 9.0%) included prostate (*n* = 103, 8.3%) and testicular cancers (*n* = 8, 0.6%). Urinary cancer (*n* = 101, 8.2%) included renal cancer (*n* = 53, 4.3%), bladder cancer (*n* = 47, 3.8%), and ureter cancer (*n* = 1, 0.1%). Head and neck cancer (*n* = 92, 7.5%) included thyroid cancer (*n* = 48, 3.9%), oral cancer (*n* = 19, 1.5%), pharyngeal cancer (*n* = 13, 1.1%), laryngeal cancer (*n* = 6, 0.5%), brain tumor (*n* = 5, 0.4%), and parotid gland cancer (*n* = 1, 0.1%). Hematological cancer (*n* = 66, 5.3%) included leukemia (*n* = 23, 1.9%), malignant lymphoma (*n* = 38, 3.1%), multiple myeloma (*n* = 4, 0.3%), myeloproliferative neoplasm (*n* = 1, 0.1%). Others (*n* = 75, 6.1%) included esophageal cancer (*n* = 20, 1.6%), hepatocellular carcinoma (*n* = 13, 1.1%), cholangiocarcinoma (*n* = 4, 0.3%), gall bladder cancer (*n* = 1, 0.1%), and pancreatic cancer (*n* = 5, 0.4%), GIST (*n* = 2, 0.2%), malignant thymoma (*n* = 3, 0.2%), skin cancer (*n* = 12, 1.0%), adrenal cancer (*n* = 1, 0.1%), various sarcoma (*n* = 10, 0.8%), and carcinoid cancer (*n* = 3, 0.2%), and cancer of unknown primary (*n* = 1, 0.1%).

**Table 1:** Basic characteristics of participants (*n* = 1,234)

	Age at cancer diagnosis, mean (SD)	Total (*n* = 1,234)	Male (*n* = 690)	Female (*n* = 544)	*p*-value
	51.45 (10.17)				
Age at cancer diagnosis (years)					
≤ 39		166	32	134	< 0.001*
40–49		325	104	221	
50–59		439	284	155	
≥ 60		304	270	34	
Time from cancer diagnosis (years)					
≤ 4	54.38 (10.14)	519	314	205	0.019*
5–9	51.25 (9.72)	413	221	192	
≥ 10	46.70 (8.93)	302	155	147	
Cancer stage					
Early (0, I)	50.88 (10.29)	711	391	320	0.447
Advanced(II-)	52.24 (9.95)	523	299	224	
Cancer site					
Intestinal	55.11 (7.91)	199	160	39	< 0.001*
Gastric	54.87 (9.01)	156	126	30	
Breast	47.08 (7.41)	230	1	229	
Female genital	40.61 (9.64)	152	0	152	
Male genital	60.64 (6.71)	111	111	0	
Lung	56.50 (7.33)	52	43	9	
Urinary	54.60 (7.82)	101	88	13	
Hematological	50.26 (10.60)	66	47	19	
Head and neck	48.38 (9.96)	92	52	40	
Other	53.55 (8.96)	75	62	13	
Surgery					
No	53.37 (10.74)	253	195	58	< 0.001*
Yes	50.96 (9.96)	981	495	486	
Chemotherapy					
No	52.05 (10.35)	817	477	340	0.014*
Yes	50.29 (9.70)	417	213	204	
Radiotherapy					
No	52.07 (10.30)	945	577	368	< 0.001*
Yes	49.43 (9.46)	289	113	176	

*p<0.05

The number of CSs with depressive symptoms (defined as HADS-depression score > 11) was 111 (9.0%) and the number of CSs with anxiety symptoms (HADS-anxiety score > 8) was 269 (21.8%). As show in Table 2, mean (SD) score of HADS-depression and HADS-anxiety were 4.08 (3.79) and 4.78 (3.79), respectively.

**Table 2:** HADS score of cancer survivors (*n* = 1,234)

	HADS-Depression			HADS-Anxiety		
	Mean (SD) score	-10	11-	Mean (SD) score	-7	8-
Total	4.08 (3.79)	1123	111	4.78 (3.79)	965	269
Age at cancer diagnosis						
≤ 39	4.69 (4.21)	149	17	5.37 (3.77)	124	42
40–49	5.02 (4.09)	293	32	5.27 (3.85)	245	80
50–59	4.70 (4.16)	393	46	4.99 (3.94)	330	109
≥ 60	3.83 (3.81)	288	16	3.64 (3.24)	266	38
Time from cancer diagnosis, years						
0–4	4.55 (4.16)	471	48	4.85 (3.83)	399	120
5–9	4.47 (4.08)	373	40	4.76 (3.80)	327	86
≥ 10	4.74 (3.95)	279	23	4.70 (3.60)	239	63
Sex						
Male	4.65 (4.13)	626	64	4.46 (3.69)	550	140
Female	4.47 (4.03)	497	47	5.20 (3.87)	415	129
Cancer stage						
Early (0, I)	4.19 (3.82)	662	49	4.46 (3.58)	577	134
Advanced (II, III, IV)	5.09 (4.37)	461	62	5.22 (4.00)	388	135
Cancer site						
Intestinal	4.01 (3.85)	185	14	4.37 (4.01)	160	39
Gastric	4.53 (4.34)	139	17	4.40 (3.81)	128	28
Breast	4.53 (4.00)	213	17	5.20 (3.72)	171	59
Female genital	4.39 (4.08)	136	16	4.87 (3.71)	125	27
Male genital	4.10 (3.54)	109	2	3.84 (3.26)	92	19
Lung	4.52 (4.40)	46	6	4.69 (4.12)	39	13
Urinary	5.03 (4.05)	89	12	5.26 (4.11)	75	26
Hematological	4.61 (3.65)	61	5	4.59 (3.02)	56	10
Head and neck	5.21 (4.37)	81	11	5.12 (3.53)	71	21
Other	5.91 (4.73)	64	11	5.80 (4.08)	48	27
Surgery						
No	4.25 (3.92)	235	18	4.54 (3.63)	205	48
Yes	4.65 (4.12)	888	93	4.85 (3.82)	760	221
Chemotherapy						
No	4.23 (3.87)	758	59	4.40 (3.60)	666	151
Yes	5.23 (4.41)	365	52	5.53 (4.03)	299	118
Radiotherapy						
No	4.45 (3.99)	863	82	4.67 (3.74)	749	196
Yes	4.95 (4.38)	260	29	5.17 (3.92)	216	73

As shown in Table 3, CSs ≥ 60 years old (reference: ≤ 39 years old, OR: 0.39, 95%CI: 0.17–0.90, *p* = 0.028), and those at least 10 years from their cancer diagnosis (reference: 0–4 years, OR: 0.55, 95%CI: 0.32–0.96, *p* = 0.037) had lower odds for depressive symptoms. CSs with gastric cancers (reference: intestinal cancer, OR: 2.25, 95%CI: 1.04–4.86, *p* = 0.039) had higher odds for depressive symptoms. In contrast, CSs with advanced (stage II, III, IV) cancers (reference: early-staged (stage 0, I), OR: 1.81, 95%CI: 1.13–2.90) had higher odds for depressive symptoms.

**Table 3:** Multivariable logistic regression analysis for depressive symptoms (HADS depression score) (*n* = 1,234)

	OR	(95%CI)	*p*-value
Age at cancer diagnosis			
≤ 39	1 (ref)		
40–49	0.97	(0.49–1.90)	0.925
50–59	0.86	(0.44–1.68)	0.652
≥ 60	0.39	(0.17–0.90)	0.028*
Time from cancer diagnosis, years			
0–4	1 (ref)		
5–9	0.91	(0.57–1.45)	0.692
≥ 10	0.55	(0.32–0.96)	0.037*
Sex			
Male	1(ref)		
Female	0.64	(0.34–1.22)	0.177
Cancer stage			
Early (0, I)	1(ref)		
Advanced (II, III, IV)	1.81	(1.13–2.90)	0.014*
Cancer site			
Intestinal	1(ref)		
Gastric	2.25	(1.04–4.86)	0.039*
Breast	1.05	(0.39–2.81)	0.923
Female genital	2.09	(0.81–5.41)	0.129
Male genital	0.29	(0.06–1.36)	0.116
Lung	1.76	(0.62–4.99)	0.287
Urinary	2.01	(0.87–4.62)	0.102
Hematological	0.96	(0.31–3.03)	0.946
Head and neck	1.56	(0.63–3.86)	0.334
Other	2.10	(0.89–4.98)	0.091
Surgery			
No	1(ref)		
Yes	1.28	(0.71–2.29)	0.415
Chemotherapy			
No	1(ref)		
Yes	1.34	(0.82–2.18)	0.244
Radiotherapy			
No	1(ref)		
Yes	1.24	(0.71–2.18)	0.454

*p<0.05

As shown in Table 4, CSs ≥ 60 years old (reference: ≤ 39 years old, OR: 0.27, 95%CI: 0.15–0.49, *p* < 0.001), and those ≥ 10 years from cancer diagnosis (reference: 0–4 years, OR: 0.62, 95%CI: 0.42–0.90, *p* = 0.011) had lower odds for anxiety symptoms. CSs who experienced chemotherapy (OR: 1.56, 95%CI: 1.10–2.20, *p* = 0.012) had higher odds for anxiety symptoms.

**Table 4:** Multivariable logistic regression analysis regarding risk factors for anxiety symptoms (HADS anxiety score) (*n* = 1,234)

	OR	(95%CI)	*p*-value
Age at cancer diagnosis			
≤ 39	1 (ref)		
40–49	0.77	(0.48–1.23)	0.268
50–59	0.71	(0.44–1.14)	0.156
≥ 60	0.27	(0.15–0.49)	< 0.001*
Time from cancer diagnosis, years			
0–4	1 (ref)		
5–9	0.75	(0.54–1.04)	0.079
10-	0.62	(0.42–0.90)	0.011*
Sex			
Male	1(ref)		
Female	1.20	(0.78–1.86)	0.408
Cancer stage			
Early (0, I)	1(ref)		
Advanced (II, III, IV)	1.27	(0.92–1.76)	0.146
Cancer site			
Intestinal	1(ref)		
Gastric	1.12	(0.64–1.97)	0.690
Breast	0.94	(0.50–1.76)	0.845
Female genital	0.58	(0.30–1.13)	0.112
Male genital	1.33	(0.69–2.59)	0.396
Lung	1.50	(0.71–3.16)	0.289
Urinary	1.61	(0.90–2.90)	0.111
Hematological	0.50	(0.22–1.13)	0.095
Head and neck	0.98	(0.52–1.88)	0.962
Other	2.19	(1.19–4.03)	0.001*
Surgery			
No	1(ref)		
Yes	1.00	(0.67–1.49)	0.988
Chemotherapy			
No	1(ref)		
Yes	1.56	(1.10–2.20)	0.012*
Radiotherapy			
No	1(ref)		
Yes	1.03	(0.70–1.51)	0.899

*p<0.05

## Discussion

As far as we know, this large-scale Japanese study, commissioned by Japanese cancer survivorship research project of the Ministry of Health, Labor and Welfare, Japan, is the first to investigate depression and anxiety symptoms among Japanese CSs. This study showed that depressive symptoms were significantly associated with three factors, namely younger age, shorter time from cancer diagnosis, and advanced cancer stage. Interestingly, anxiety symptoms, while also significantly associated with younger age and shorter time from cancer diagnosis, was not associated with advanced cancers, but rather experience of chemotherapy.

Our study showed that younger CSs tended to have more depressive and anxiety symptoms than older survivors (≥ 60 years old), in line with several previous studies [13–15]. In contrast, Mols et al. reported that older age was also associated with depressive symptoms, but with fewer symptoms of anxiety [16]. Nonetheless, most studies suggest that depressive symptoms among CSs appear to decrease with increased age [14]. Firstly, younger CSs might choose more aggressive treatments than older patients [17], while older CSs have more medical comorbidities than younger patients. Secondly, younger CSs may experience greater difficulty with social role conflicts and returning to work, and harbor more concerns about reproductive health, sleep difficulties, and financial problems, and therefore experience greater psychological distress [13, 18]. Sutin reported that younger CSs may experience intense social stressors but may lack the necessary coping mechanisms associated with the accumulated experience and wisdom of older persons [15]. As such, younger CSs may need more support than older patients as they may be more prone to suffer from the cancer-related stress [19].

Regarding time from cancer diagnosis, depressive and anxiety symptoms were reported to be highest when closest to the time of cancer diagnosis, a finding also in line with previous studies [20, 21].Burgess et al. reported that over time, depressive and anxiety symptoms among cancer patients tended to improve [22], though Ruijs et al. reported that depression and anxiety increase in CSs who are about to die [23]. As the subjects of our study did not include CSs who are nearing death, it is reasonable to expect that depressive and anxiety symptoms tend to improve with increased distance from diagnosis. While previous studies suggest that average quality of life among CSs does not differ from that of the general population after 5 years from cancer diagnosis [24], long-term cancer survivorship does not imply that psychological or physical recovery is complete [13]. Longitudinal studies of depression after cancer diagnosis have suggested that the high initial prevalence of depression and anxiety falls slowly over time [25]. Mitchell’s meta-analysis reported that the relative risks of depression decreased with time after cancer diagnosis [26]. Mols et al. reported that HADS-depression scores changed over time, while HADS-anxiety scores did not [16]. A meta-analysis by Swartzman et al. reported that longer durations since cancer diagnosis were associated with incremental decreases in post-traumatic stress disorder on the order of 0.5% for each additional year after cancer diagnosis [27]. Nonetheless, some evidence suggests that frequency of depression and anxiety does not decrease considerably over time [28].

Our study confirms the finding that more advanced cancers are associated with depression. As CSs with advanced cancers may experience relatively high level of distress regardless of age [20], cancer stage (poor-prognosis cancers) can have a considerable influence on the prevalence of depression and anxiety [29]. Despite the possibility of complete remission, especially for particular malignancies such as testicular cancer, advanced cancers generally indicate both more intensive therapeutic courses and poorer prognoses [13]. Patients who are closer to death report higher existential distress but do not show greater risk for adjustment or anxiety disorders [30]. Walker et al. reported that greater depression and anxiety were strongly associated with worse subsequent survival for both sexes among most types of CSs, coupled with the fact that depression and anxiety are highly associated [3]. Dunn et al. reported that cancer stage predicted high psychological distress in colorectal CSs for up to several years after their diagnosis [31]. However, it should be noted that depressive symptoms are often complicated by cancer-related somatic symptoms (weakness, pain, and fatigue) that make diagnosis more difficult [29]. A meta-analysis by Janberidze et al. reported that patients with advanced cancer had a higher prevalence of depression than patients with early-stage cancer [32]. The potential mechanisms for this association have been proposed, but none proven [33].

Results of our study showed that experience of chemotherapy was also a risk factor for anxiety, but not for depression. Chemotherapy causes a variety of adverse effects, affecting quality of life in all of its domains, including psychological distress, persistent fatigue, cognitive impairment, sexual dysfunction, infertility, neuropathy, and hair loss, among a host of other untoward symptoms [34, 35]. Anxiety can become a pathologic disorder when it is excessive and uncontrollable, requires no specific external stimulus, and manifests with a wide range of physical and affective symptoms as well as changes in behavior and cognition [36]. Many previous studies have report that cognitive function declines occur in CSs who received chemotherapy and chemotherapy-induced cognitive impairment has been reported to be associated with depression and anxiety [37, 38]. A longitudinal study by Ho et al. reported that depression and anxiety within one year of chemotherapy were extremely prevalent [39]. However, Mystakidou et al. reported that, among advanced-staged CSs, depression has a significant correlation with chemotherapy, with a lower correlation shown for anxiety [40].

### Clinical Implications and Study Limitations

This study has several limitations that warrant discussion. Firstly, the study design allows for the possibility of recall bias, which may in itself be affected by changes in cognitive function due to cancer treatments. As some CSs may not have a clear memory of objective events or subjective feelings around the time of cancer diagnosis, longitudinal, prospective studies of the mental well-being of CSs, as they undergo treatment and beyond, are needed. Secondly, this study may be affected by survivorship bias, a form of selection bias due to non-inclusion of cancer patients who died before completing the questionnaire. While our study is by definition limited to survivors, we acknowledge that the full breath of mental health outcomes may not be captured in this study population. Thirdly, relevant risk factors for anxiety and depression, such as comorbidities, poor cognitive function, socioeconomic risk factors such as employment and economic stress, and caregiving status for young children may not be fully represented in our sample [18, 19]. Finally, we excluded CSs who had already received a psychiatric diagnosis or treatment, which could limit the generalizability of our results to patients with more severe symptoms. Due to these limitations, caution is warranted when applying our findings to the general cancer setting.

## Conclusion

In conclusion, younger CSs, those with recent cancer diagnoses, advanced-staged cancers, and undergoing chemotherapy may be at higher risk for poor mental health outcomes. Patients in these demographic groups warrant particularly careful follow-up by medical professionals in order to optimize mental health and well-being, and provide adequate and timely support for psychiatric illness.

## Data Availability

The data that support the findings of this study could be available on request from the corresponding author. The data are not publicly available due to privacy or ethical restrictions.

## Abbreviations

CSs: Cancer Survivors
HADS: The Hospital Anxiety and Depression Scale
